# Supplementary material for: YTH-RNA-binding protein prevents deleterious expression of meiotic proteins by tethering their mRNAs to nuclear foci
Source: eLife. 2018 Feb 9;7:e32155. doi: 10.7554/eLife.32155 (PMC5807050; doi:10.7554/eLife.32155)
Supplement: Supplementary file 1. [file elife-32155-supp1.docx]

**Supplementary file 1. Strains used in this study.**

| Strain name | Genotype | Figure |  |
| --- | --- | --- | --- |
| JS53 | *h90 ade6-M216 leu1 mei4-4xTAP-mei4UTR::natR* | 1B, 1C, 6E, 6F |  |
| JS54 | *h90 ade6-M216 leu1 mei4-4xTAP-mei4UTR::natR mmi1-ts3-kanR* | 1B, 1C, 4E, 5C, 6E, 6F, 6-s2 |  |
| JS55 | *h90 ade6-M216 leu1 mei4-4xTAP-mei4UTR::natR red1::bsdR* | 1B, 1C, 6-s2 |  |
| JS56 | *h90 ade6-M216 leu1 mei4-4xTAP-mei4UTR::natR red1::bsdR mmi1-ts3-kanR* | 1B, 1C |  |
| JS57 | *h90 ade6-M216 leu1 mei4-4xTAP-mei4UTR::natR rrp6-32-bsdR* | 1B, 1C |  |
| JS58 | *h90 ade6-M216 leu1 mei4-4xTAP-mei4UTR::natR rrp6-32-bsdR mmi1-ts3-kanR* | 1B, 1C |  |
| JS59 | *h90 ade6-M216 leu1 red1::bsdR* | 1A, 1-s2A, 1-s2B, 1-s2C, 1-s2D, 2A, 2B, 2-s1A, 2-s1B |  |
| JS60 | *h90 ade6-M216 leu1 red1::bsdR mmi1-ts3-kanR* | 2A, 2B, 2-s1A, 2-s1B |  |
| JS61 | *h90 ade6-M216 leu1 rrp6-32-bsdR* | 1A, 1-s2A, 1-s2B, 2A, 2B, 2-s1A, 2-s1B |  |
| JS62 | *h90 ade6-M216 leu1 rrp6-32-bsdR mmi1-ts3-kanR* | 1-s2A, 1-s2B, 2A, 2B, 2-s1A, 2-s1B |  |
| JS63 | *h90 ade6-M216 leu1 ura4-D18 red1::bsdR mei4::ura4^+^* | 1A |  |
| JS64 | *h90 ade6-M216 leu1 ssm4-3GFP-kanR mmi1-ts3-bsdR* | 1-s1A, 1-s1B, 4-s1E, 5-s3, 6-s1D, 6-s1E |  |
| JS65 | *h90 ade6-M216 leu1 ssm4-3GFP-kanR red1::hphR* | 1-s1A, 1-s1B |  |
| JS66 | *h90 ade6-M216 leu1 ssm4-3GFP-kanR red1::hphR mmi1-ts3-bsdR* | 1-s1A, 1-s1B |  |
| JS67 | *h90 ade6-M216 leu1 ssm4-3GFP-kanR rrp6-32-hphR* | 1-s1A, 1-s1B |  |
| JS68 | *h90 ade6-M216 leu1 ssm4-3GFP-kanR rrp6-32-hphR mmi1-ts3-bsdR* | 1-s1A, 1-s1B |  |
| JS69 | *h90 ade6-M216 leu1 rae1-167-bsdR* | 2C, 2D, 2-s1C, 2-s1D |  |
| JS70 | *h90 ade6-M216 leu1 rae1-167-bsdR mmi1-ts3-kanR* | 2C, 2D, 2-s1C, 2-s1D |  |
| JS71 | *h90 ade6-M210 leu1 CO2::Padh1-4xU1A-Luc-natR arg1::Padh41-U1Ap-YFP-hphR* | 3B, 3C, 3-s1A |  |
| JS72 | *h90 ade6-M210 leu1 CO2::Padh1-4xU1A-Luc-4xTTAAAC-natR arg1::Padh41-U1Ap-YFP-hphR* | 3C, 3-s1A, 3-s1B |  |
| JS73 | *h90 ade6-M210 leu1 CO2::Padh1-4xU1A-Luc-8xTTAAAC-natR arg1::Padh41-U1Ap-YFP-hphR* | 3C, S3-s1A, 3-s1B |  |
| JS74 | *h90 ade6-M210 leu1 CO2::Padh1-4xU1A-Luc-10xTTAAAC-natR arg1::Padh41-U1Ap-YFP-hphR* | 3C, 3-s1A, 3-s1B |  |
| JS75 | *h90 ade6-M210 leu1 CO2::Padh1-4xU1A-Luc-12xTTAAAC-natR arg1::Padh41-U1Ap-YFP-hphR* | 3C, 3-s1A, 3-s1B |  |
| JS76 | *h90 ade6-M210 leu1 CO2::Padh1-4xU1A-Luc-14xTTAAAC-natR arg1::Padh41-U1Ap-YFP-hphR* | 3B, 3C, 3D, 3-s1A, 3-s2C |  |
| JS77 | *h90 ade6-M210 leu1 CO2::Padh1-4xU1A-Luc-16xTTAAAC-natR arg1::Padh41-U1Ap-YFP-hphR* | 3C, 3-s1A, 3-s1B |  |
| JS78 | *h90 ade6-M210 leu1 CO2::Padh1-4xU1A-Luc-18xTTAAAC-natR arg1::Padh41-U1Ap-YFP-hphR* | 3C, 3-s1A, 3-s1B |  |
| JS79 | *h90 ade6-M210 leu1 CO2::Padh1-4xU1A-Luc-20xTTAAAC-natR arg1::Padh41-U1Ap-YFP-hphR* | 3C, 3-s1A, 3-s1B |  |
| JS80 | *h90 ade6-M210 leu1 CO2::Padh1-4xU1A-Luc-22xTTAAAC-natR arg1::Padh41-U1Ap-YFP-hphR* | 3C, 3-s1A, 3-s1B |  |
| JS81 | *h90 ade6-M210 leu1 CO2::Padh1-4xU1A-Luc-24xTTAAAC-natR arg1::Padh41-U1Ap-YFP-hphR* | 3B, 3C, 3D, 3-s1A, 3-s2C, 5-s2C |  |
| JS82 | *h90 ade6-M210 leu1 ura4-D18 CO2::Padh1-4xU1A-Luc-14xTTAAAC-natR arg1::Padh41-U1Ap-YFP-hphR LEU2-CFP-mmi1* | 3E |  |
| JS83 | *h90 ade6-M210 leu1 mei4::kanR CO2::Padh1-4xU1A-Luc-14xTTAAAC-natR arg1::Padh41-U1Ap-YFP-hphR* | 3-s2A, 3-s2B, 3-s2C |  |
| JS84 | *h90 ade6-M216 leu1 ura4-D18 mei4::ura4^+^ mmi1::kanR CO2::Padh1-4xU1A-Luc-14xTTAAAC-natR arg1::Padh41-U1Ap-YFP-hphR* | 3-s2A, 3-s2B, 3-s2C |  |
| JS85 | *h90 ade6-M216 leu1 red1::bsdR CO2::Padh1-4xU1A-Luc-14xTTAAAC-natR arg1::Padh41-U1Ap-YFP-hphR* | 3-s2A, 3-s2B, 3-s2C, 3-s2D |  |
| JS86 | *h90 ade6-M216 leu1 red1::bsdR CO2::Padh1-4xU1A-Luc-14xTTAAAC-natR arg1::Padh41-U1Ap-YFP-hphR LEU2-CFP-mmi1* | 3-s2E |  |
| JS87 | *h90 ade6-M216 leu1 rrp6-32-bsdR CO2::Padh1-4xU1A-Luc-14xTTAAAC-natR arg1::Padh41-U1Ap-YFP-hphR* | 3-s2A, 3-s2B, 3-s2C, 3-s2D |  |
| JS88 | *h90 ade6-M216 leu1 rrp6-32-bsdR CO2::Padh1-4xU1A-Luc-14xTTAAAC-natR arg1::Padh41-U1Ap-YFP-hphR LEU2-CFP-mmi1* | 3-s2E |  |
| JS89 | *h90 ade6-M216 leu1 CO2::Padh1-4xU1A-Luc-24xTTAAAC-natR arg1::Padh41-U1Ap-YFP-hphR LEU2-CFP-mmi1* | 3E |  |
| JS90 | *h90 ade6-M216 leu1 mei4::kanR CO2::Padh1-4xU1A-Luc-24xTTAAAC-natR arg1::Padh41-U1Ap-YFP-hphR* | 3F, 3G, 3-s2C, 5-s2C |  |
| JS91 | *h90 ade6-M216 leu1 ura4-D18 mei4::ura4^+^ mmi1::kanR CO2::Padh1-4xU1A-Luc-24xTTAAAC-natR arg1::Padh41-U1Ap-YFP-hphR* | 3F, 3G, 3-s2C, 5-s2A, 5-s2B, 5-s2C |  |
| JS92 | *h90 ade6-M216 leu1 red1::bsdR CO2::Padh1-4xU1A-Luc-24xTTAAAC-natR arg1::Padh41-U1Ap-YFP-hphR* | 3F, 3G, 3-s2C, 3-s2D |  |
| JS93 | *h90 ade6-M210 leu1 red1::bsdR CO2::Padh1-4xU1A-Luc-24xTTAAAC-natR arg1::Padh41-U1Ap-YFP-hphR LEU2-CFP-mmi1* | 3-s2E |  |
| JS94 | *h90 ade6-M216 leu1 rrp6-32-bsdR CO2::Padh1-4xU1A-Luc-24xTTAAAC-natR arg1::Padh41-U1Ap-YFP-hphR* | 3F, 3G, 3-s2C, 3-s2D |  |
| JS95 | *h90 ade6-M216 leu1 rrp6-32-bsdR CO2::Padh1-4xU1A-Luc-24xTTAAAC-natR arg1::Padh41-U1Ap-YFP-hphR LEU2-CFP-mmi1* | 3-s2E |  |
| JS96 | *h90 ade6-M210 leu1 ura4-D18 mmi1::kanR mei4::ura4^+^* | 4B, 4-s1A |  |
| JS97 | *h90 ade6-M210 leu1 CO2::Padh1-3HA-mmi1(FL)-natR* | 4D, 6D |  |
| JS98 | *h90 ade6-M210 leu1 CO2::Padh1-3HA-mmi1(∆YTH)-natR* | 4D |  |
| JS99 | *h90 ade6-M216 leu1 CO2::Padh1-3HA-mmi1(∆SID)-natR* | 4D |  |
| JS100 | *h90 ade6-M216 leu1 kanR-TAP-mmi1 CO2::Padh1-3HA-mmi1(FL)-natR* | 4D, 6D |  |
| JS101 | *h90 ade6-M216 leu1 kanR-TAP-mmi1 CO2::Padh1-3HA-mmi1(∆YTH)-natR* | 4D |  |
| JS102 | *h90 ade6-M216 leu1 kanR-TAP-mmi1 CO2::Padh1-3HA-mmi1(∆SID)-natR* | 4D |  |
| JS103 | *h90 ade6-M210 leu1 red1-13myc-kanR CO2::Padh1-3HA-mmi1(FL)-natR* | 4G, 6-s1C |  |
| JS104 | | *h90 ade6-M216 leu1 red1-13myc-kanR CO2::Padh1-3HA-mmi1(∆YTH)-natR* | 4G |
| JS105 | | *h90 ade6-M216 leu1 red1-13myc-kanR CO2::Padh1-3HA-mmi1(∆SID)-natR* | 4G |
| JS106 | | *h90 ade6-M216 leu1 erh1::kanR LEU2-CFP-mmi1* | 6A |
| JS107 | | *h90 ade6-M216 leu1 red1::bsdR natR-CFP-mmi1* | 6-s1A |
| JS108 | | *h90 ade6-M216 leu1 ura4-D18 rhn1::ura4^+^ LEU2-CFP-mmi1* | 6-s1A |
| JS109 | | *h90 ade6-M216 leu1 iss9::kanR LEU2-CFP-mmi1* | 6-s1A |
| JS110 | | *h90 ade6-M216 leu1 rhn1-mCherry-hphR LEU2-CFP-mmi1* | 6-s1B |
| JS111 | | *h90 ade6-M216 leu1 erh1-YFP-kanR LEU2-CFP-mmi1* | 6B |
| JS112 | | *h90 ade6-M216 leu1 iss9-YFP-kanR LEU2-CFP-mmi1* | 6-s1B |
| JS113 | | *h90 ade6-M216 leu1 erh1-GFP-kanR CO2::Padh1-3HA-mmi1(FL)-natR* | 6C |
| JS114 | | *h90 ade6-M210 leu1 erh1-GFP-kanR CO2::Padh1-3HA-mmi1(∆YTH)-natR* | 6C |
| JS115 | | *h90 ade6-M216 leu1 erh1-GFP-kanR CO2::Padh1-3HA-mmi1(∆SID)-natR* | 6C |
| JS116 | | *h90 ade6-M216 leu1 erh1::hphR CO2::Padh1-3HA-mmi1(FL)-natR* | 6D |
| JS117 | | *h90 ade6-M216 leu1 erh1::hphR kanR-TAP-mmi1 CO2::Padh1-3HA-mmi1(FL)-natR* | 6D |
| JS118 | | *h90 ade6-M216 leu1 erh1::hphR red1-13myc-kanR CO2::Padh1-3HA-mmi1(FL)-natR* | 6-s1C |
| JS119 | | *h90 ade6-M216 leu1 erh1::kanR CO2::Padh1-4xU1A-Luc-24xTTAAAC-natR arg1::Padh41-U1Ap-YFP-hphR* | 5-s2A, 5-s2B, 5-s2C |
| JS121 | | *h90 ade6-M210 leu1 ura4-D18 erh1::hphR mei4::ura4^+^* | 6G |
| JS122 | | *h90 ade6-M216 leu1 mei4-4xTAP-mei4UTR::natR erh1::kanR* | 6E, 6F |
| JS123 | | *h90 ade6-M210 leu1 erh1::hphR ssm4-3GFP-kanR* | 6-s1D, 6-s1E |
| JS174 | | *h90 ade6-M216 leu1 mei4-4xTAP-mei4UTR::natR pab2::kanR* | 6-s2 |
| JS175 | | *h90 ade6-M216 leu1 ura4-D18 mmi1-ts3-kanR red1::ura4^+^* | 1-s2A, 1-s2B |
| JT645 | | *h90 ade6-M216 leu1 ssm4-3GFP-kanR* | 1-s1A, 1-s1B, 6-s1D, 6-s1E |
| JT764 | | *h90 ade6-M216 leu1 rrp6-32-kanR mei4::hphR* | 1A |
| JT958 | | *h90 ade6-M216 leu1 erh1::hphR* | 5A, 5B, 5-s1A, 5-s1B, 6G |
| JV579 | | *h90 ade6-M216 leu1 mmi1-ts3-kanR* | 1-s2A, 1-s2B, 1-s2C, 1-s2D, 2A, 2B, 2-s1A, 2-s1B, 4F, 5A, 5B , 5-s1A, 5-s1B |
| JV862 | | *h90 ade6-M216 leu1 LEU2-CFP-mmi1* | 6A |
| JY362 | | *h+/h- ade6-M210/ade6-M216 leu1/leu1* | 1-s2C, 1-s2D |
| JY450 | | *h90 ade6-M216 leu1* | 1A, 1-s2A, 1-s2B, 2A, 2B, 2-s1A, 2-s1B 6G |
| JZ768 | | *h90 ade6-M216 leu1 ura4-D18 mei4::ura4^+^* | 1A, 6G |
