# Supplementary material for: YTH-RNA-binding protein prevents deleterious expression of meiotic proteins by tethering their mRNAs to nuclear foci
Source: eLife. 2018 Feb 9;7:e32155. doi: 10.7554/eLife.32155 (PMC5807050; doi:10.7554/eLife.32155)
Supplement: Supplementary file 2. [file elife-32155-supp2.docx]

**Supplementary file 2. Primers used in this study.**

| Primer name | Sequence | Purpose |
| --- | --- | --- |
| act1 F | TGAGGAGCACCCTTGCTTGT | qRT-PCR in figure 1B,1-s1A, 4E, 4-s1E, 6E, 6-s1D |
| act1 R | TCTTCTCACGGTTGGATTTGG |  |
| mei4 F | AACCAAATGCTGAAACTCAAGAA | qRT-PCR in figure 1B, 4E, 6E |
| mei4 R | CGTTGACGTTTTCATAAAGGCTA |  |
| ssm4 F | AACAGCTAAAGACCGCAAGG | qRT-PCR in figure 1-s1A, 4-s1E, 6-s1D |
| ssm4 R | TCTCCTTGCAGGCAAAGGTC |  |
| U1A F | ATGCGTCGACCGGGTACCGAGCTCGAA | Construction of the DNA fragment to probe reporter transcripts in figure 3-s1A, 3-s2C, 5-s2C |
| U1A R | GATCCTCGAGACTAGGATCTGCCAATTG |  |
| mei4 probe U | ATGGTTGAAAATCAAGGGAATG | Construction of the DNA fragment to probe reporter transcripts in figure 1-s2A, 1-s2C |
| mei4 probe L | GATCGATCTAATACGACTCACTATAGGGCTATCTTGATTTGATGGTTGAC |  |
| ssm4 probe U | TGTATGGCAAGTTGACAACG | Construction of the DNA fragment to probe reporter transcripts in figure 1-s2B, 1-s2D |
| ssm4 probe L | GATCGATCTAATACGACTCACTATAGGGTAATTCCTCTTGAGAGTCTG |  |
