# Supplementary material for: YTH-RNA-binding protein prevents deleterious expression of meiotic proteins by tethering their mRNAs to nuclear foci
Source: eLife. 2018 Feb 9;7:e32155. doi: 10.7554/eLife.32155 (PMC5807050; doi:10.7554/eLife.32155)
Supplement: Supplementary file 3. [file elife-32155-supp3.docx]

**Supplementary file 3. Oligonucleotide probes for single molecule FISH.**

| Gene name | Sequence |
| --- | --- |
| *mei4* | TTCGACATTCCCTTGATTTT  AGTATGACCTTTTTGGGTTT  CTTCAGTTTCGCATTTTTCT  GGTTTTCTTTATTCTCTTCA  TGTCGTATACTGTCACTACT  GCCATTTCATCTCCAAATAT  TTCACCAGTATCCACAAAGC  TAGCATAAGAACACGGTGGT  TAGGATCGCCAAACCGATTA  GTCAGTTGCTTGTTATGTGA  AATCCACGTATAAATCCCGC  ATCATGGTTGAGGTAGTAGC  GTTGTGCCTAATGCTATTTT  AAAGTTTTACCCTTGGGTTT  AAGTTCTGCATATGGTCAGG  GAACGATGTAAGCGAACGCT  ACTGTTTGAGTCAGTTGAGT  ATTTGCTTGAAGGTCGCTTT  TCGTCAACGGTTTGATTTCA  CGAGCTAATGGTATCTCTCG  AGAGTTTAATCGACTTCGCT  CGAGCCAGAAGTTGATGTTG  CTTCGGCAGCAACATTTGAA  TTGATGGTTGACTTGCATCG  ATTAGAAGGGGGAAGAGGGG  GGTTTAGGTACATTTTCTGA  GATCTTCTTGAGTTTCAGCA  TAAAGGCTAGACTCATGGGC  GACGAGAATCGTTGACGTTT  CCCAGTATTTAATGCCATAT  TAACCAGGATCTGCATCAGA  CTGTTATGGCTATTAGAGCG  CTTCATTGGCAGAATACGGA  AATCTGCCTGTAGAACATCC  GCATGGAATGAGGATCTCGA  ATGGGTTCCCTACGATAATA  AACGGGATGATGGACGTAGT  GTTGGGCGTGTATATTCATA  AAGAGGTATCTGTACGACCG  GTTGAACAAAAAGCGCCGGG  GGACTTGAAGGTGCACATTT  CATGTTCGCGATGTTTTTGT  ATCCATCGAAAGAGGGTGTT  TGACTTGAAGGATTCCACGG  CAAACAAAGGCTCCGAGAGC  GTCCCTAAAACATGTTACCG  TAGCCACTTGACTCATGATT  ACGCTCGATTAGAAGGCATT |
| *ssm4* | CCCGACGGACAAATAACTCA  CTAATTCTCCACGGATGAGG  TCAGTACTACCGGCAAATCG  GCCTAACCAAATACCACTTT  CATCATTTTTTCCTTTTCCA  AAATATCCCTTTTCCTTTCT  TCACATTCGAAGAGCATGCG  CTTGACGACACTTGGTCTTT  TTTCTGATCCTTTTTTCCTT  CCCTACGTGATTTTTGTTTA  TGTAATGCACAATGGGCGTG  TGTCTTTGTGGGAGTAGACG  ATTTGCTCGTATCTGAGTTT  AAACTACTATCGTCAAGCCC  TGCCATACAAATTCCTCTTC  TTTTTTTTCACATTCCTGCA  ACTTCCCTTTGAATGAGGAG  CCTTCCTTAATTCTGACTTT  AGGCGGTATTTTCACACATC  ACTGAGGTTCAACTTTCTCT  GCCTAAAATTCCGAGGTTTT  ATTTCTTTCTGCATAGCCAA  GCAACTTTCGCTTTCTTAGA  TGGCTTTTAGGAGTTTTGGA  TGTAACTTGTGTAGTCCAGT  CTTAGAAACCTCTGAGACCC  CTCAGTTATGTCGACCTGAA  AAATCCAAACTTCCCGGTAC  CTGTGCTATTCCAATGTACA  TCCACTGAAGAGTTGTTGGA  CAACAAAGCATTTTCCTCCA  TCTTGCTTCTATTTCAGTGT  CACCTCATTTAACTTTTCCG  ACTGAGATAGCCTTTGAGTT  CTTCTGTCAAAGCAGGTTGA  ATTCCTCTTGAGAGTCTGAT  TTGCTTTCTCAAGTTTTGCA  TTGCACTGTTTAACTCGTCT  TCCTCCACTTGTTTTGATAG  AGGTCGTCTTTCATACTACT  ATATTTATCGGCCAGTTTCA  CACTAGTATTGTCACCTTGT  AAACTGTGTTTGAGCCAGTC  GGGCTTGGAATTGAGTTTTC  ATTTTCTTATCTCCTTGCAG  GCTTTTCTACTGAAACGGGT  TTTTTCCAATTCAAGGCCAG  GATGAAAACTGGGTCGCTCG |
