# Supplementary material for: YTH-RNA-binding protein prevents deleterious expression of meiotic proteins by tethering their mRNAs to nuclear foci
Source: eLife. 2018 Feb 9;7:e32155. doi: 10.7554/eLife.32155 (PMC5807050; doi:10.7554/eLife.32155)
Supplement: Source data 1. [file elife-32155-data1.docx]

# Supplementary file 4. Uncropped images of western and northern blots.

**Figure 1C**

*WT*

*red1∆*

*rrp6-32*

# Figure 1-figure supplement 2A

*red1∆*

*rrp6-32*

*mmi1-ts3 red1∆ mmi1-ts3 rrp6-32 mmi1-ts3*


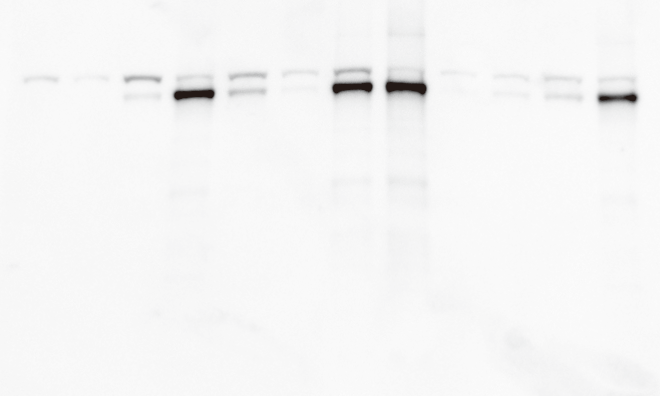


*WT mmi1-ts3 red1∆ mmi1-ts3 rrp6-32 mmi1-ts3*

(kDa) 150

102

76

52

38

31

25 37 25 37 30 37 30 37 25 37 25 37 (°C)

Mei4-TAP

*mei4*

(°C)

25 37 25 37 30 37 30 37 25 37 25 37

150


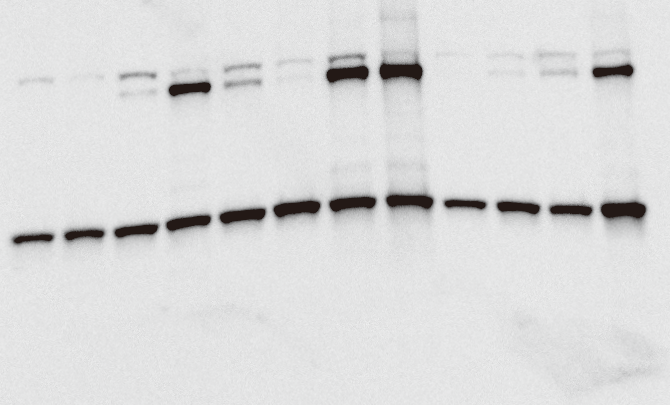

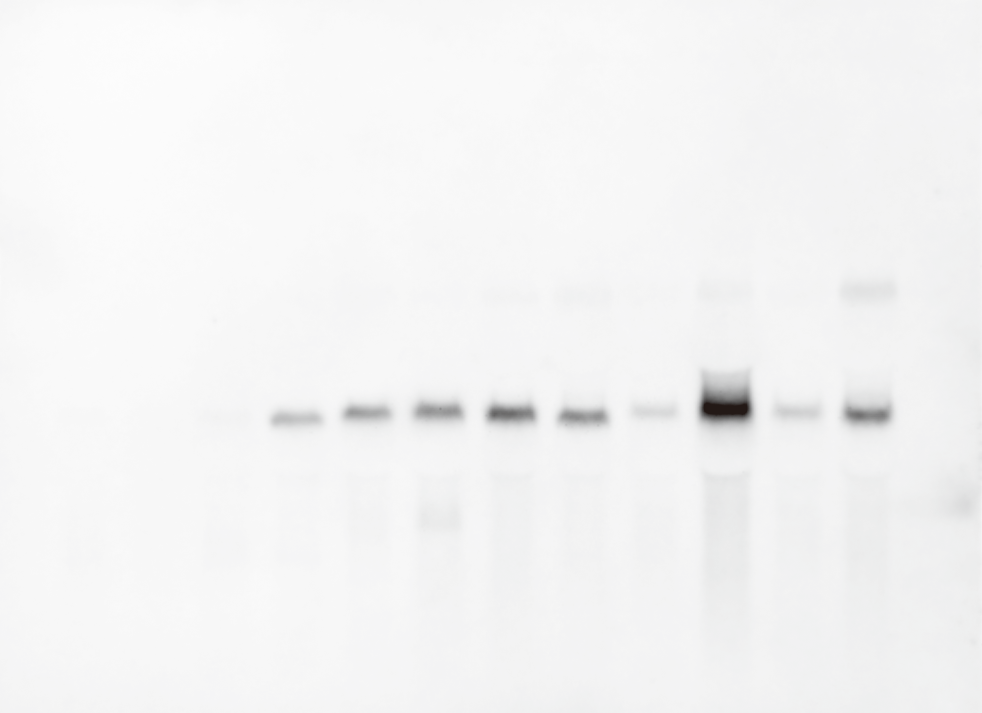


102

76

γ-Tubulin

52

38

31

# Figure 1-figure supplement 1B

**Figure 1-figure supplement 2B**

*WT*

*red1∆*

*rrp6-32*

*red1∆*

*rrp6-32*

*mmi1-ts3 red1∆ mmi1-ts3 rrp6-32 mmi1-ts3*

*WT mmi1-ts3 red1∆ mmi1-ts3 rrp6-32mmi1-ts3*

(°C)


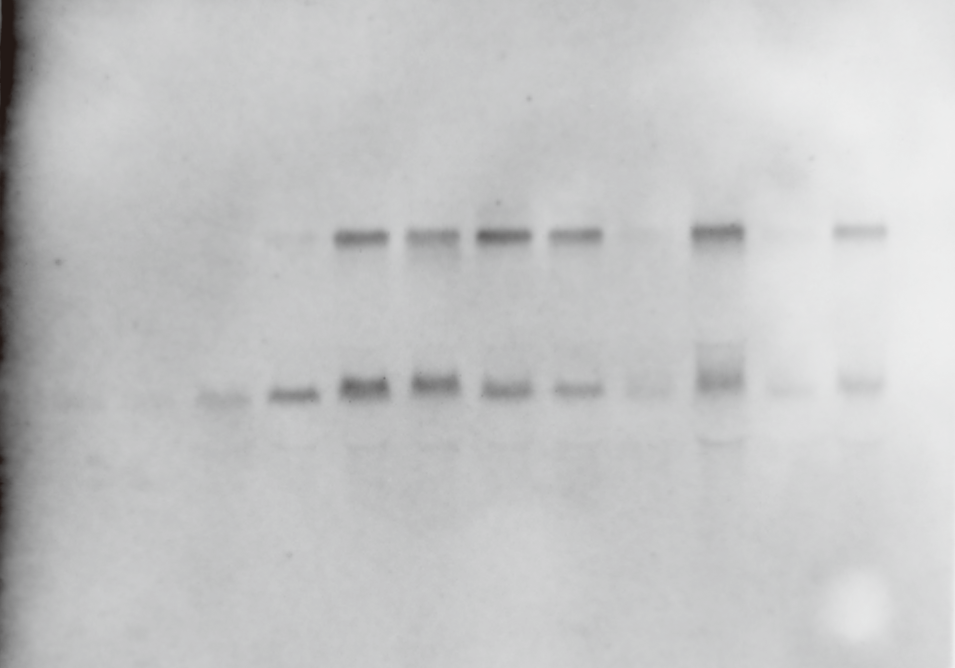


25 37 25 37 30 37 30 37 25 37 25 37

(°C)

25 37 25 37 30 37 30 37 25 37 25 37

Ssm4-3GFP


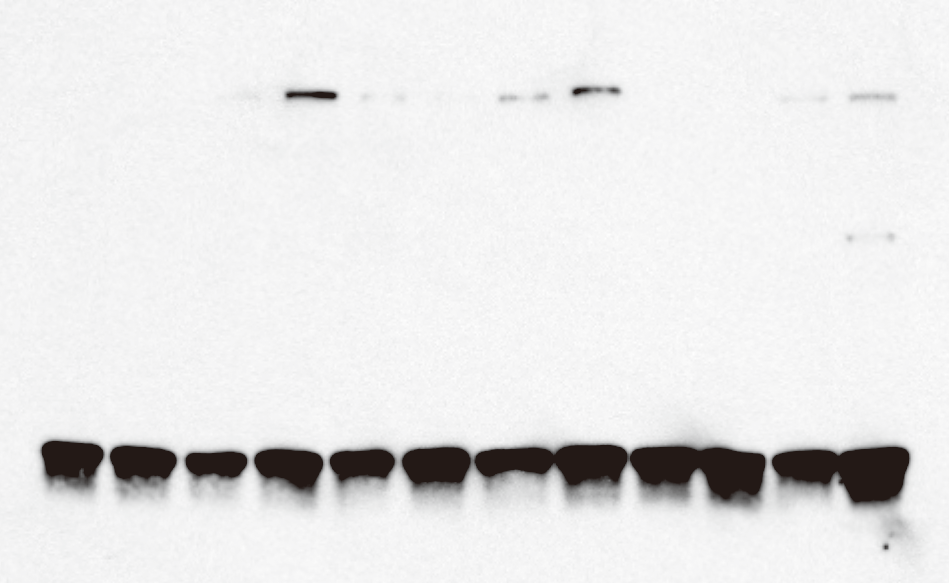


*ssm4*

γ-Tubulin


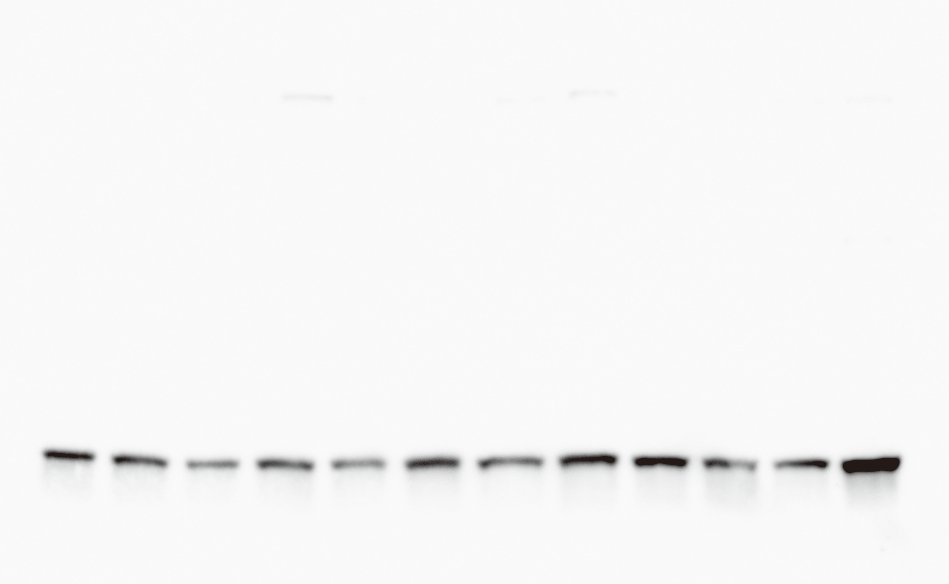


# Figure 1-figure supplement 2C Figure 3-figure supplement 1A

*WT mmi1-ts3 red1∆*

(xTTAAAC)

0 4 8 10 12 14 16 18 20 22 24

(°C) -N 25 37 30 37


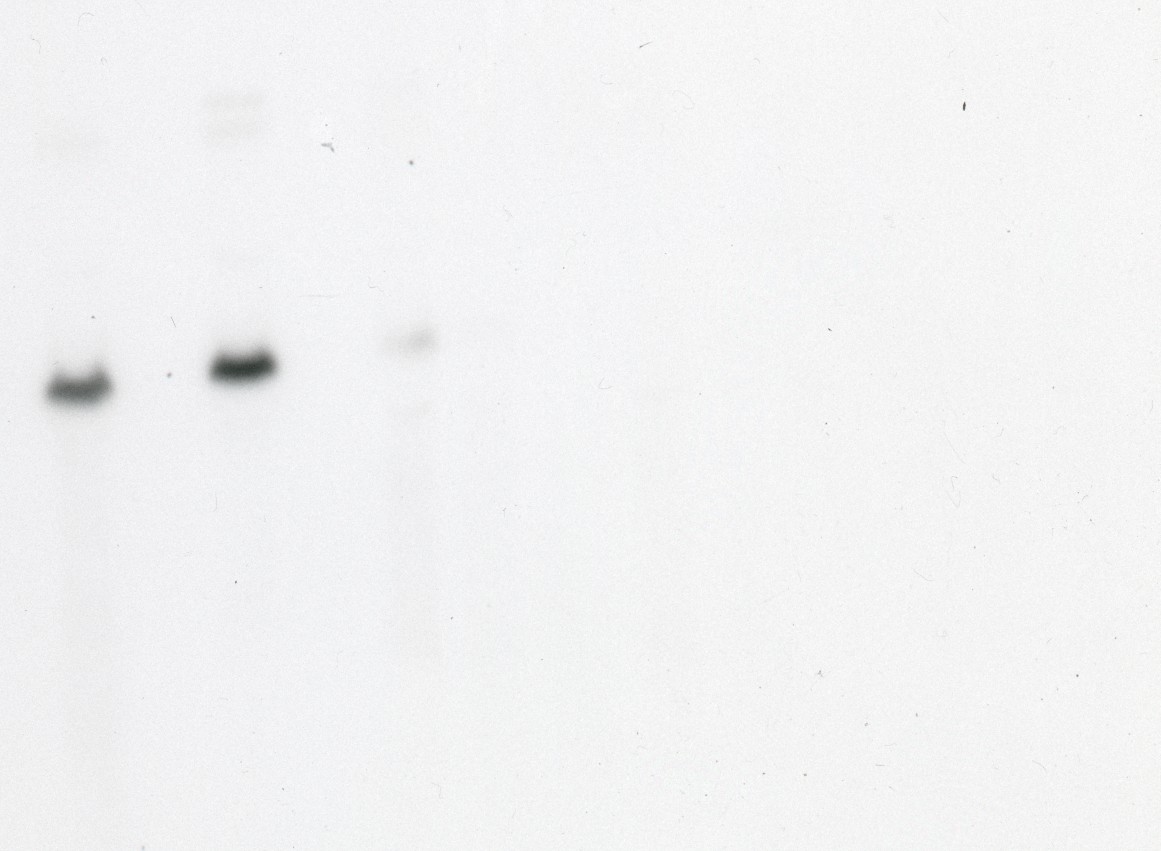

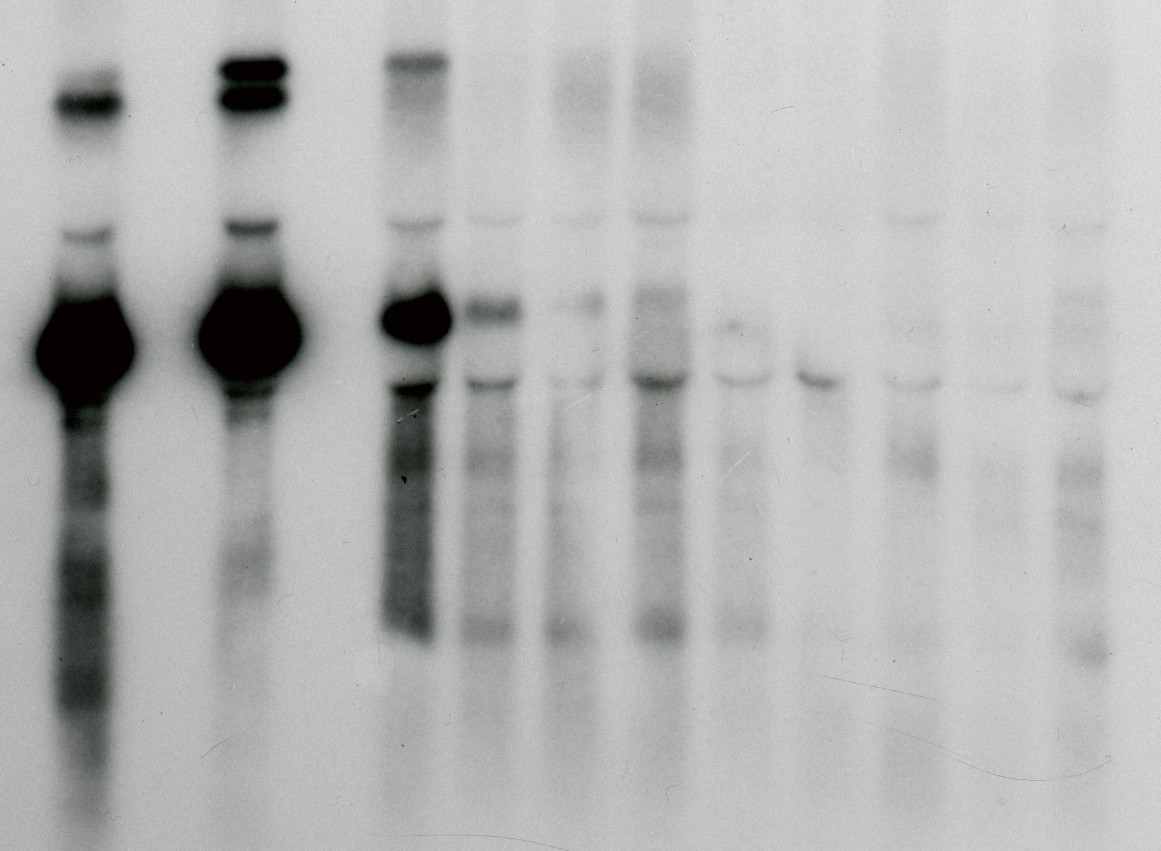


U1A


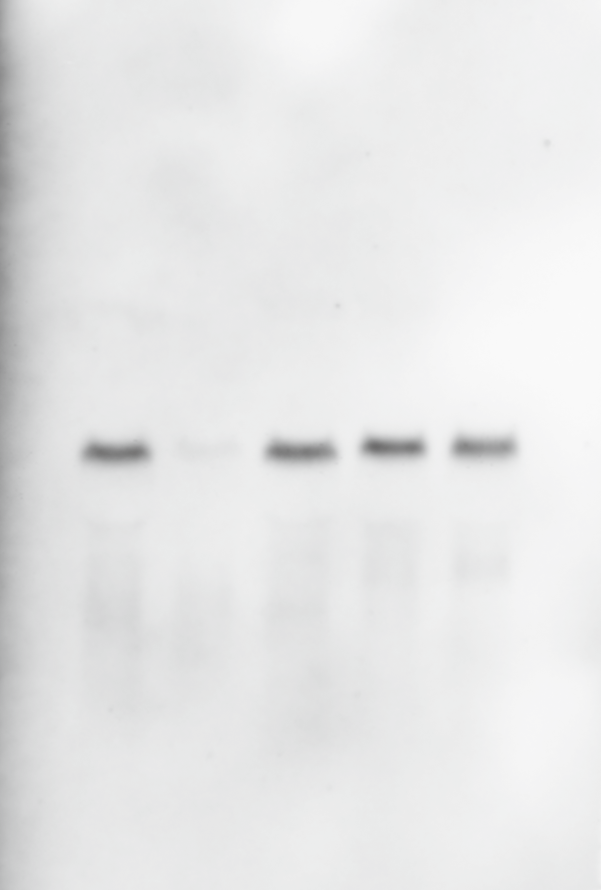


*mei4*

(long exposure)

(kb) 2.0

1.0

0.5

# Figure 1-figure supplement 2D Figure 3-figure supplement 2C

14x 24x

*WT mmi1-ts3 red1∆*

*WT*

*mei4∆ mmi1∆ mei4∆ red1∆*

*WT*

*mei4∆ mmi1∆ mei4∆ red1∆*

(°C) -N

25 37 30 37

*rrp6-*

*32*

25 37

*rrp6-*

*32*

25 37

U1A


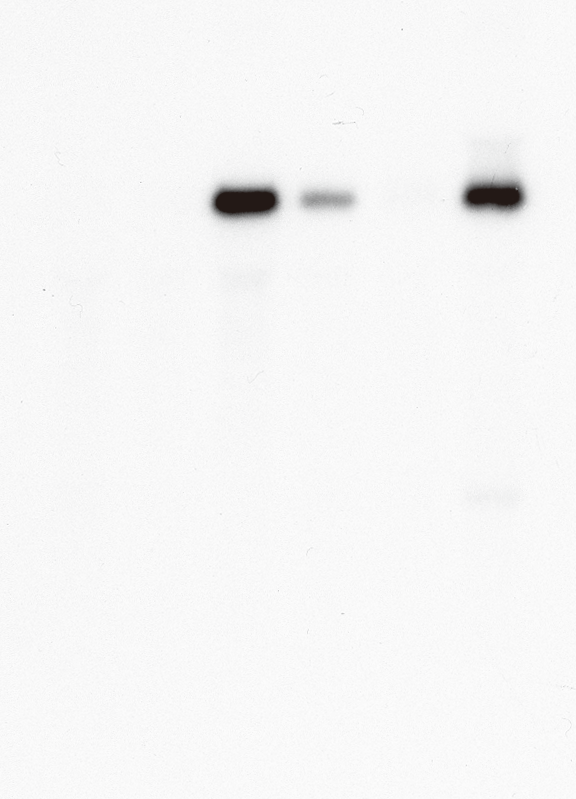

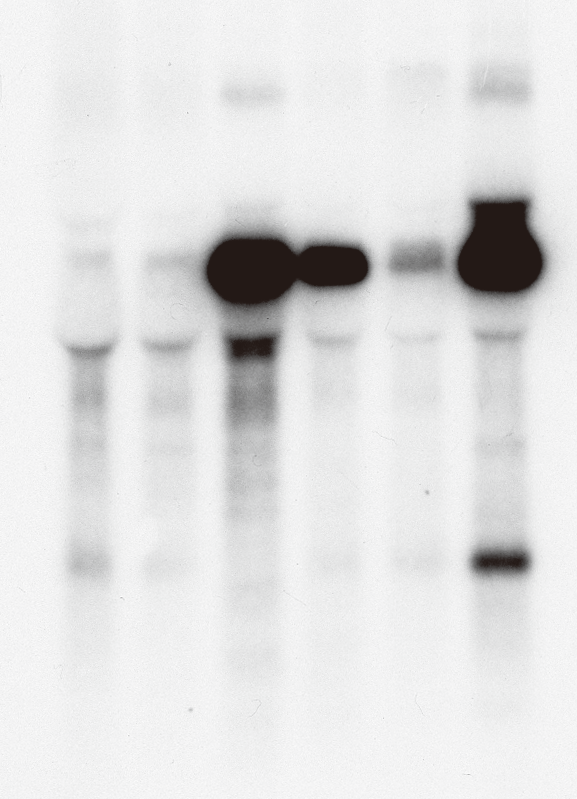

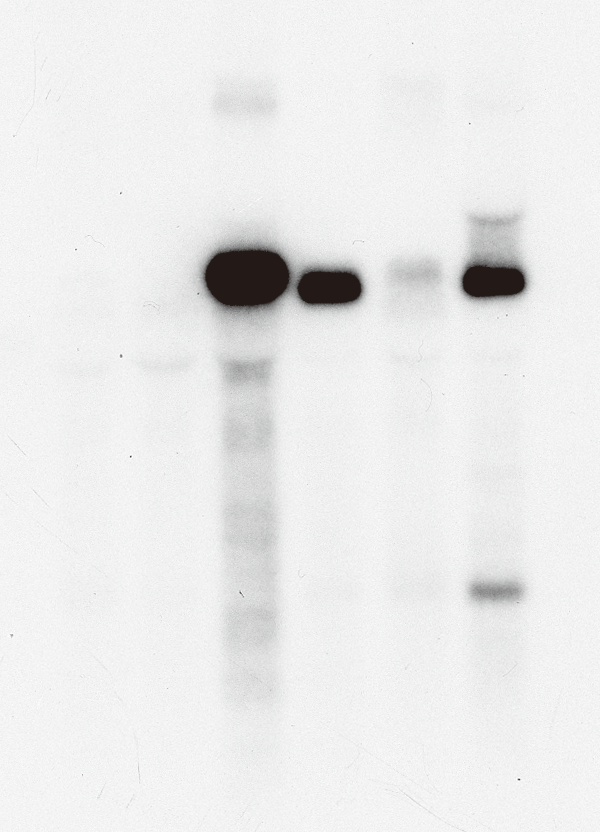

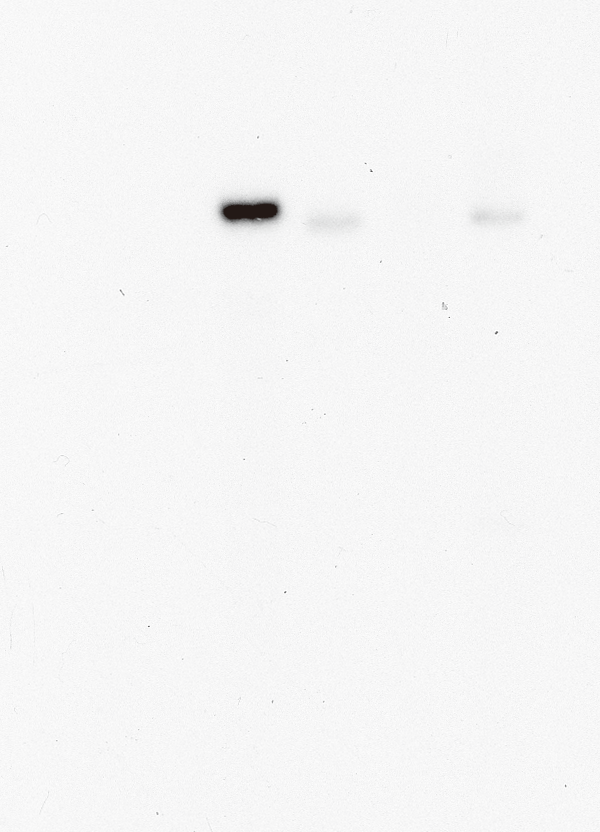

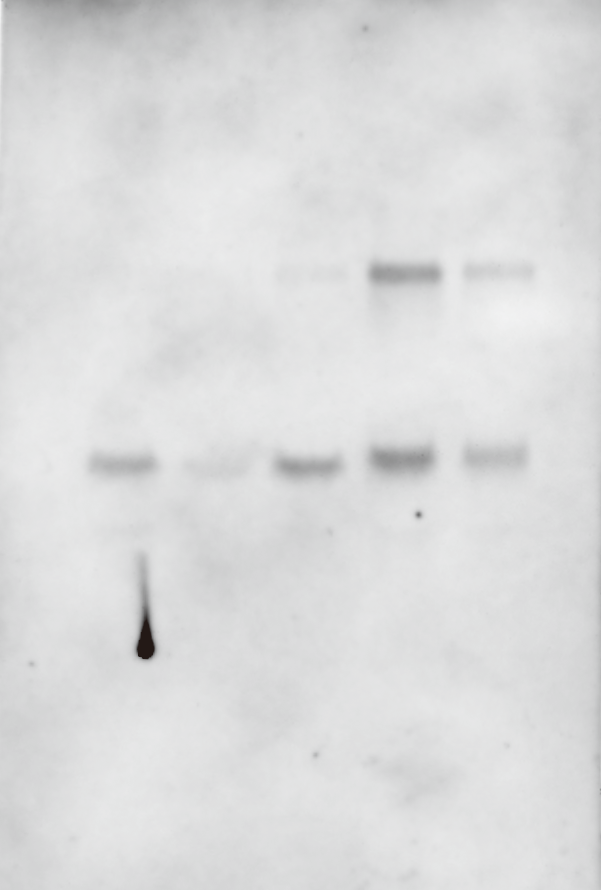


*ssm4*

(long exposure)

# Figure 4D Figure 4-figure supplement 1D

1/10 WCE IP (mouse IgG)

*TAP- TAP-*

FL

∆YTH

∆SID

FL

∆YTH

∆SID

FL

∆YTH

∆SID

FL

∆YTH

∆SID

(kDa) 150

102

76

52

38

31

## 150

102

76

52

38

31

*mmi1+ mmi1 mmi1+*

*mmi1*

3HA-Mmi1


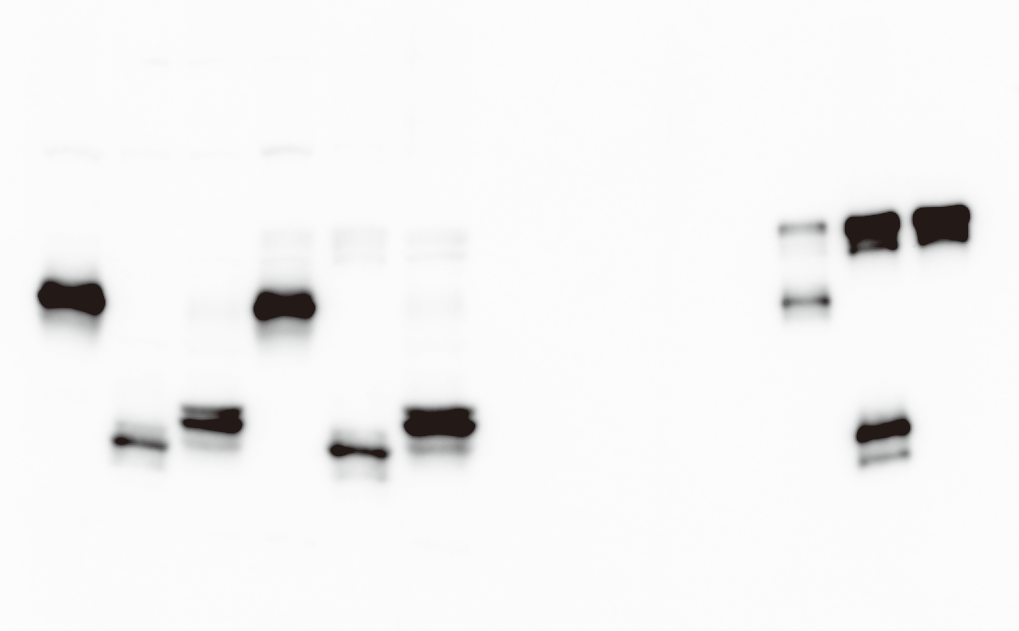

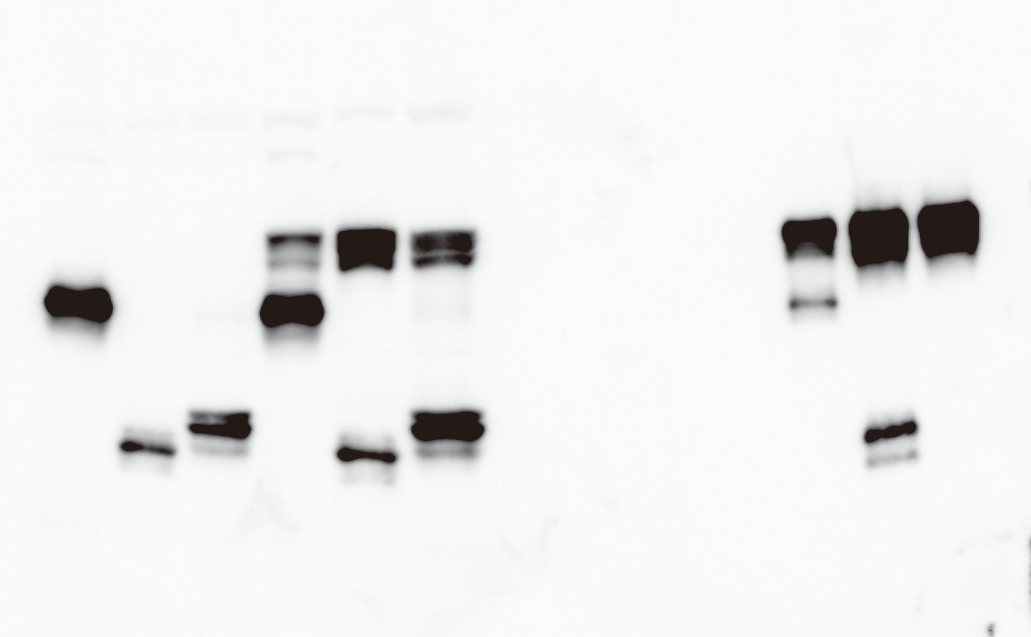


3HA-Mmi1

TAP-Mmi1

GST-Mmi1

GST FL ∆YTH ∆SID


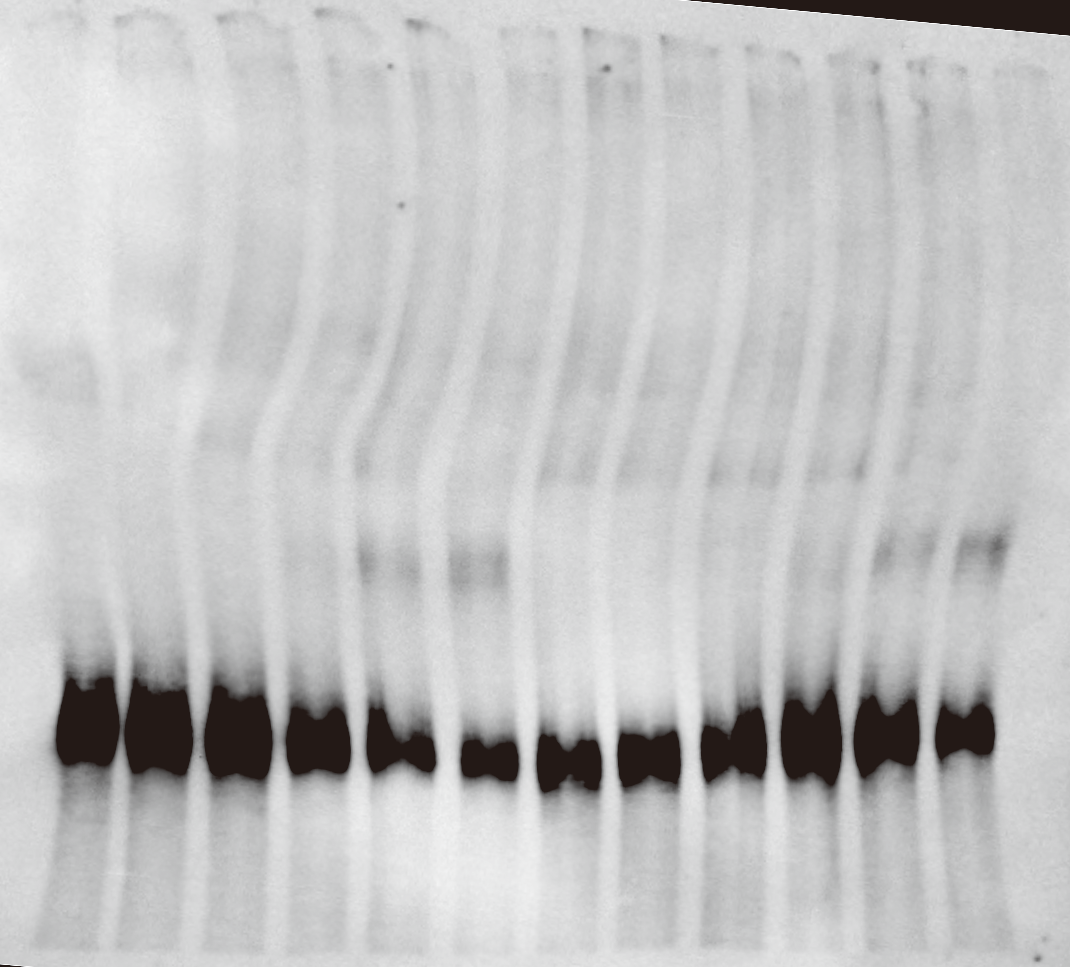


# Figure 4G

1/10

IP (anti- IP

# Figure 5C

*mmi1-ts3*

FL

∆YTH

∆SID FL

∆YTH

∆SID

(kDa) 225

FL

∆YTH

∆SID

150

102

76

52

38

31

24

225

150

102

76

52

38

31

24

WCE

DDDDK) (anti-HA)

3HA-Mmi1


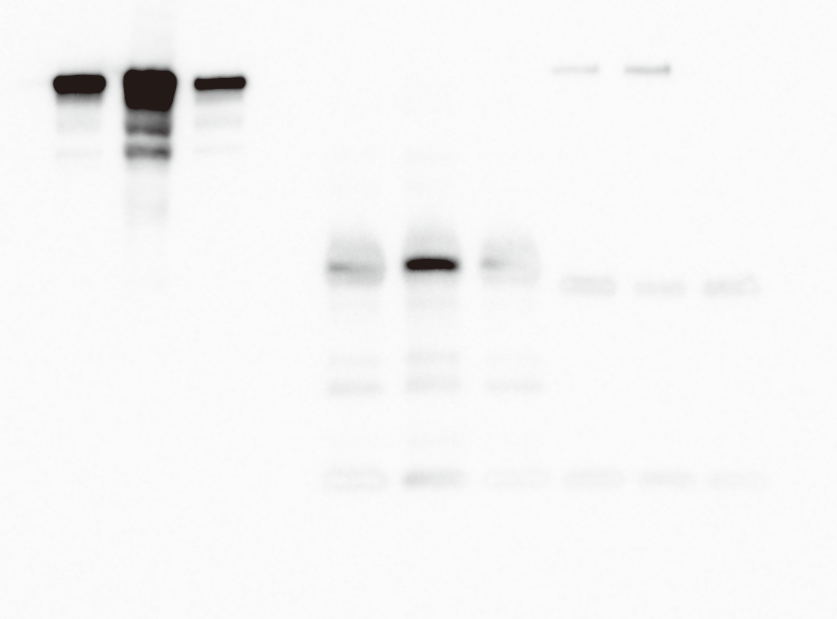


Red1-13Myc

3HA-Mmi1


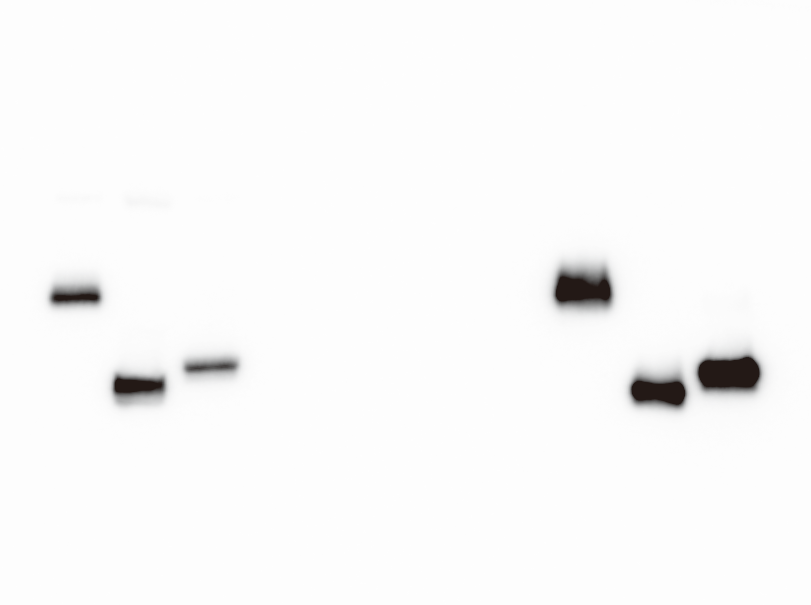


(kDa) 150

102

76

52

38

31

## 150

102

76

52

38

31

vector *FL ∆YTH ∆SID*

25 37 25 37 25 37 25 37 (°C)


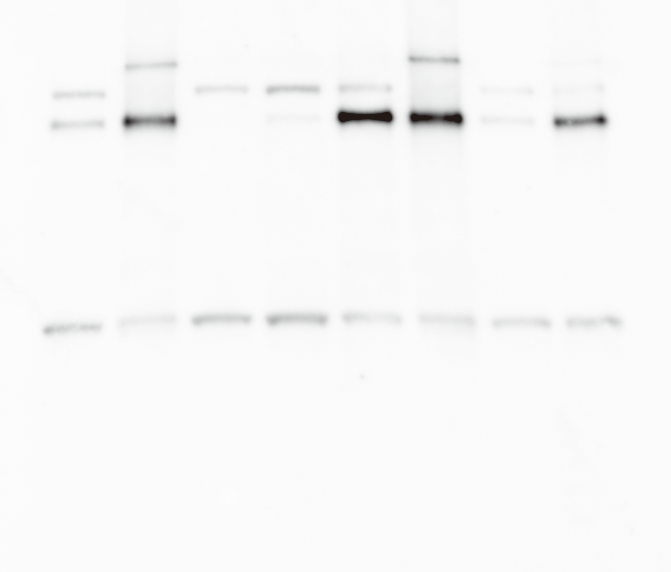


Mei4-TAP

γ-Tubulin


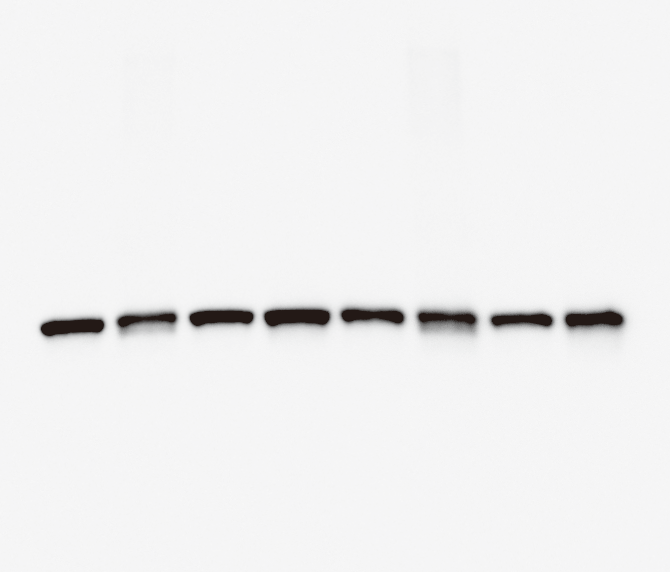


# Figure 5-figure supplement 2C

*WT*

*mei4∆ mmi1∆ mei4∆ erh1∆*

*mmi1∆*

# Figure 6C

1/10

IP (anti- IP

*mei4∆*


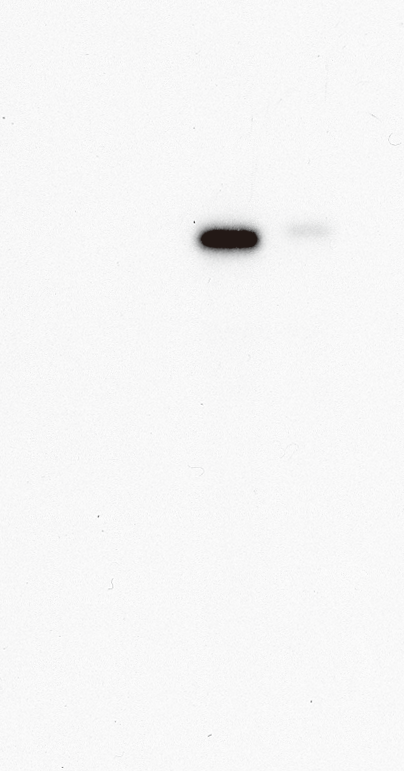


vector

*FL*

*∆YTH*

*∆SID*

WCE

DDDDK) (anti-HA)

FL

∆YTH

∆SID

U1A


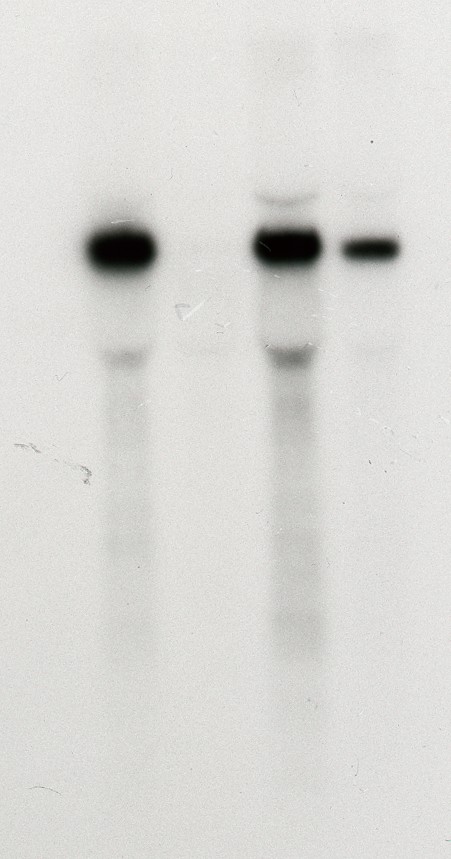


(long exposure)


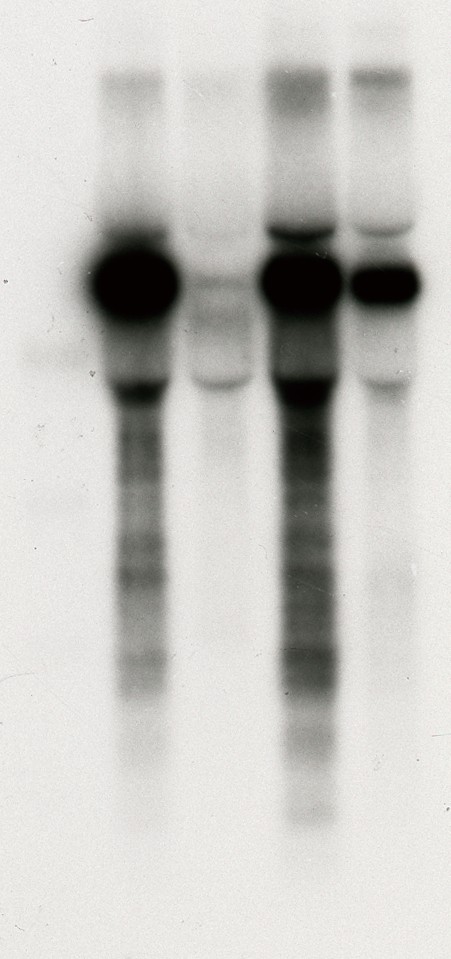


(kDa) 150

102

76

52

38

31

## 150

102

76

52

38

31

3HA-Mmi1

Erh1-GFP


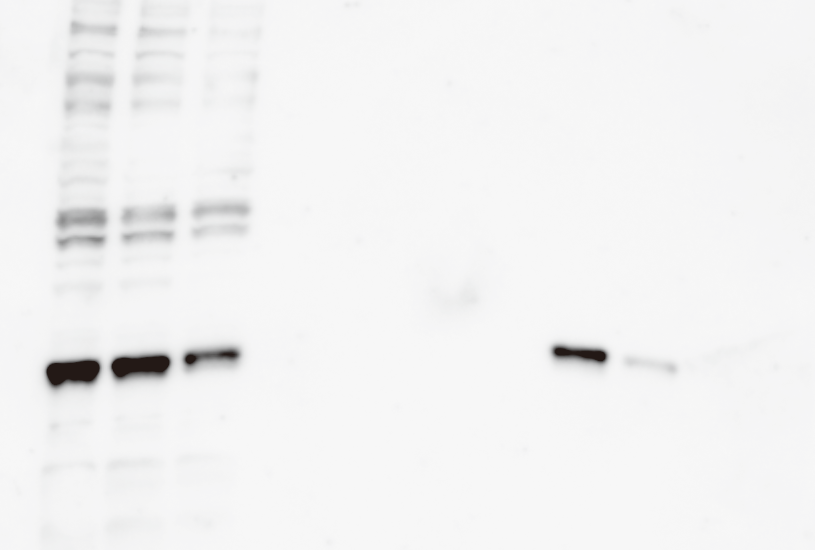

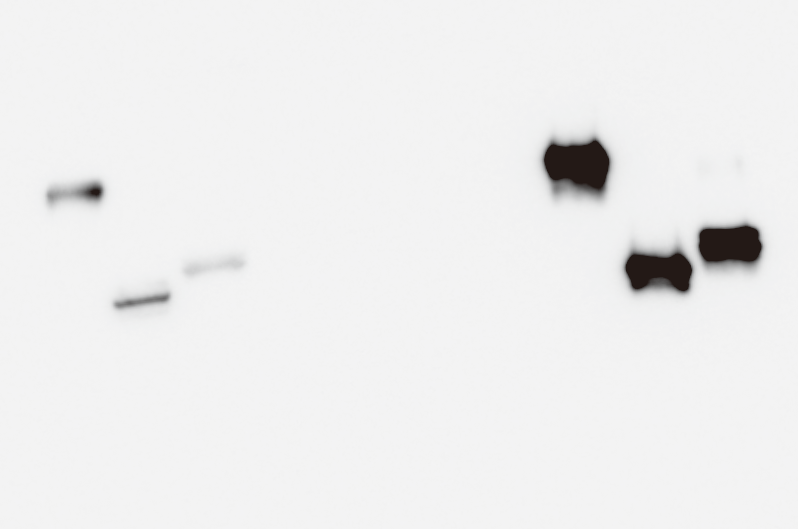


FL

∆YTH

∆SID FL

∆YTH

∆SID

3HA-Mmi1

# Figure 5-figure supplement 3


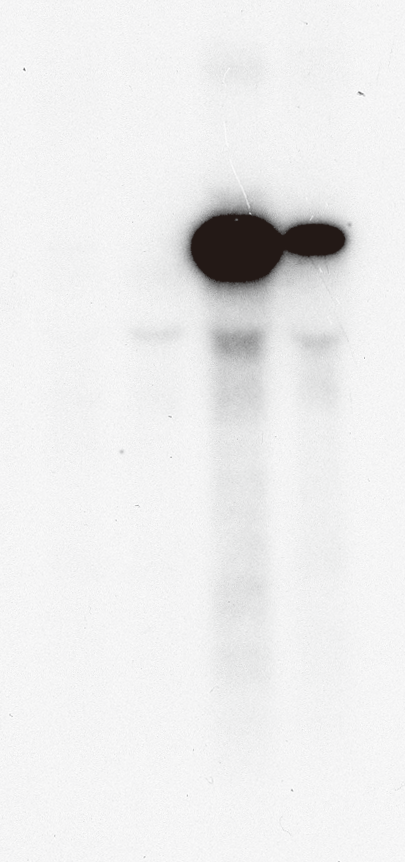


*mmi1-ts3*

# Figure 6D

1/10 WCE

*TAP-*

IP (IgG)

*TAP-*

vector *FL ∆YTH ∆SID*

*mmi1+ mmi1 mmi1+ mmi1*

(kDa)

225


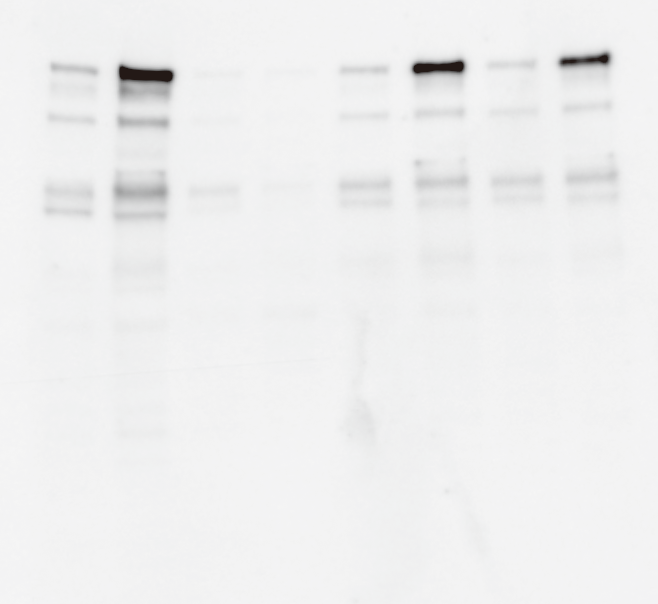


150

102 76

52

38

31

24

225


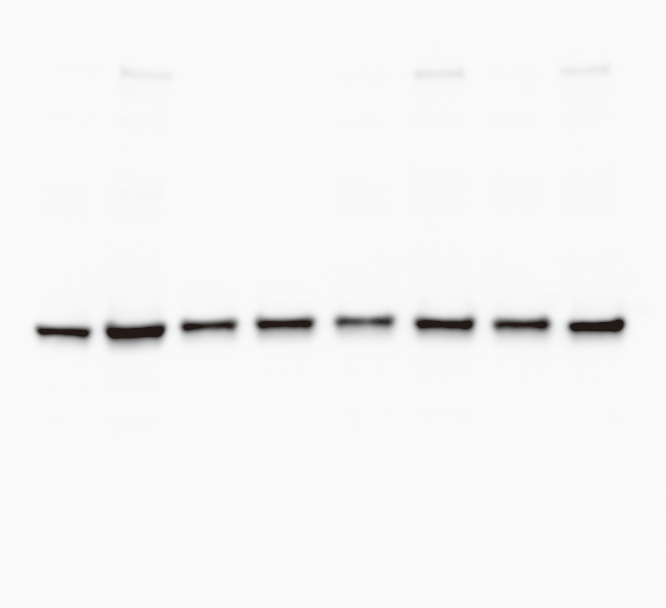


150

102

76

52

38

31

24

25 37 25 37 25 37 25 37 (°C)

Ssm4-3GFP

γ-Tubulin

(kDa) **150**

102

76

52

38

31

## 150

102

76

52

38

31

+ ∆ + ∆ + ∆ + ∆

*erh1*

TAP-Mmi1


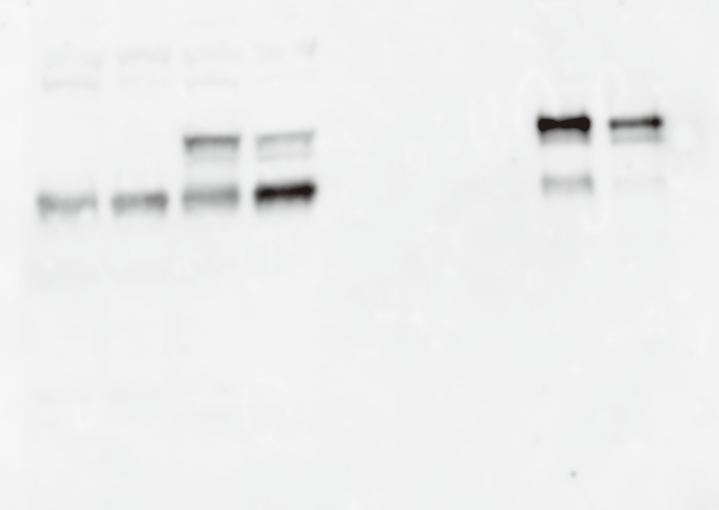

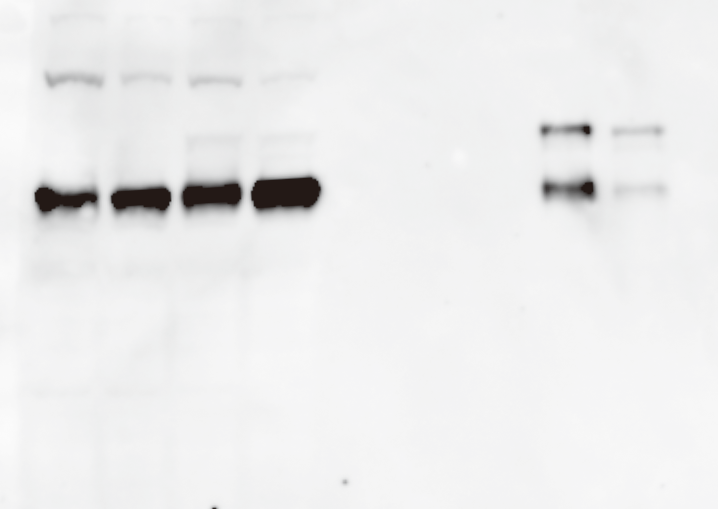


3HA-Mmi1

# Figure 6F

*erh1∆*

*mmi1*

*WT*

*-ts3*

30 25 37 30 (°C)

(kDa)


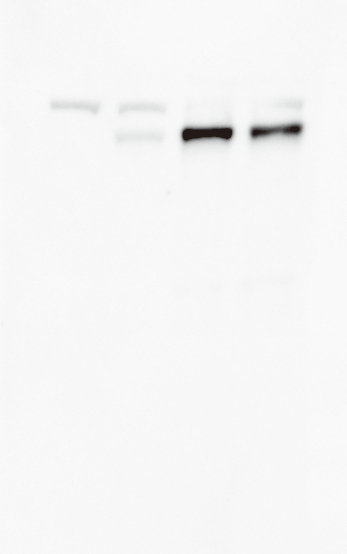


150

102

76

52

38

31

Mei4-TAP

# Figure 6-figure supplement 1E

*mmi1*

*WT*

*erh1∆*

*-ts3*

30 25 37 30 (°C)


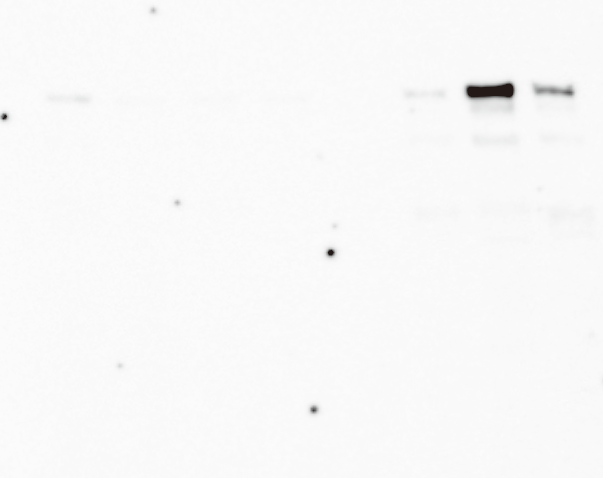


Ssm4-3GFP

150


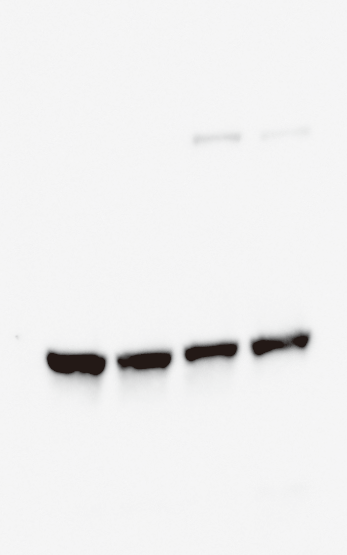

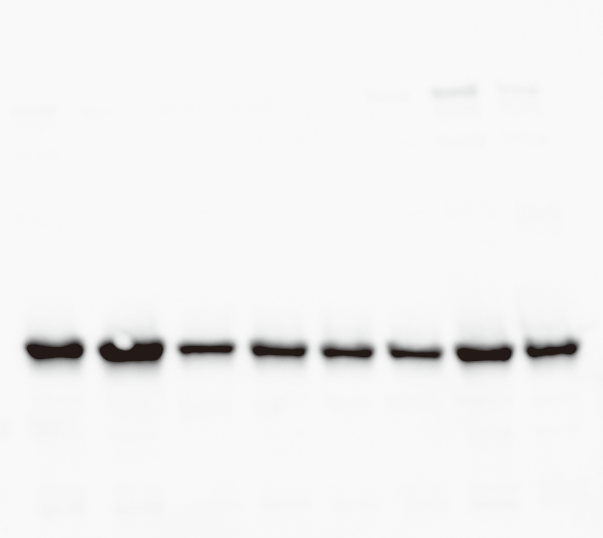


102

76

52 γ-Tubulin

38

31

γ-Tubulin

# Figure 6-figure supplement 1C

**Figure 6-figure supplement 2**

*mmi1-ts3 red1∆ pab2∆*

(kDa) 150

102

76

52

38

31

## 150

102

76

52

38

31

1/10

WCE

IP(anti- DDDDK)

IP

(anti-HA)

+ ∆ + ∆ + ∆

*erh1*


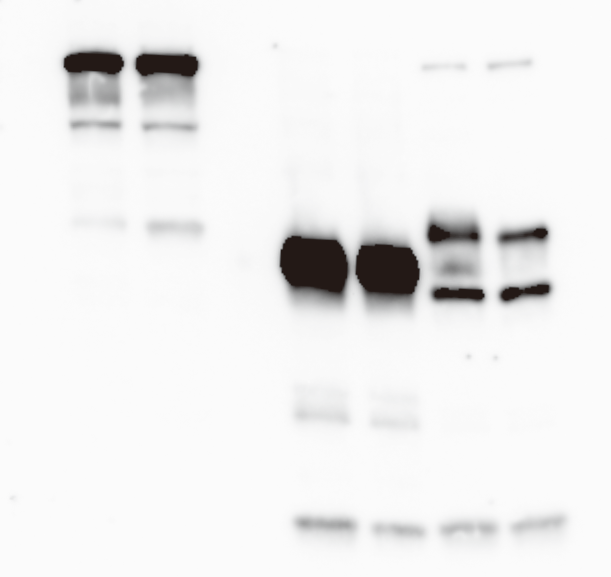

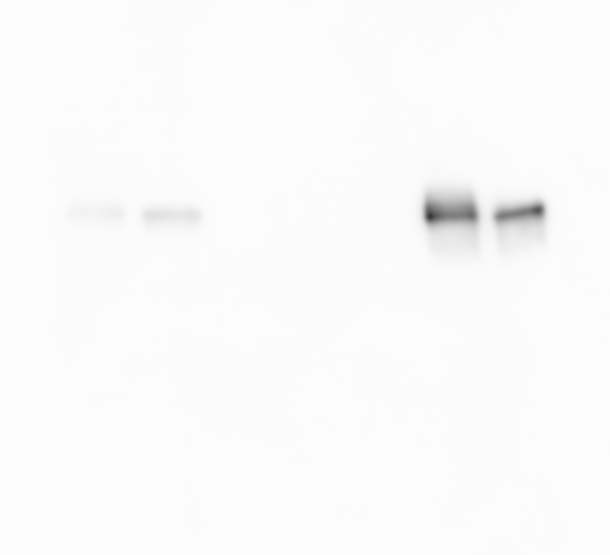


Red1-13Myc

3HA-Mmi1

(°C)

(kDa) 150


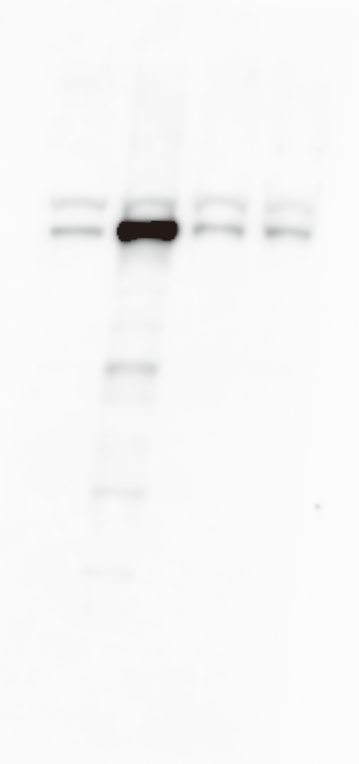


102

76

52

38

31

25 37 30 30

Mei4-TAP

(kDa) 150


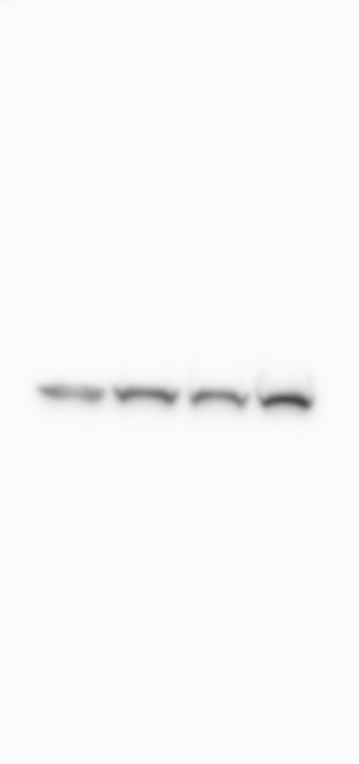


102

76

52 γ-Tubulin

38

31
